# Music, health, and well-being: A review

**DOI:** 10.3402/qhw.v8i0.20635

**Published:** 2013-08-07

**Authors:** Raymond A. R. MacDonald

**Affiliations:** School of Music, Edinburgh College of Art, University of Edinburgh, Edinburgh, UK

**Keywords:** Music health and well-being, psychology, therapy community, music

## Abstract

The relationship between arts participation and health is currently very topical. Motivated by a desire to investigate innovative, non-invasive, and economically viable interventions that embrace contemporary definitions of health, practitioners and researchers across the world have been developing and researching arts inventions. One of the key drivers in this vigorous research milieu is the growth of qualitative research within health care contexts and researchers interested in exploring the potential benefits of musical participation have fully embraced the advances that have taken place in health-related qualitative research. The following article presents a number of different types of qualitative research projects focused on exploring the process and outcomes of music interventions. It also presents a new conceptual model for music, health and well-being. This new model develops on a previous version of MacDonald, Kreutz, and Mitchell (2012b) by incorporating new elements and contextualization and providing detailed experimental examples to support the various components.

The relationship between arts participation and health has received significant and growing academic, media, and public attention over the past 10 years. Motivated by a desire to investigate innovative, non-invasive, and economically viable interventions that embrace contemporary definitions of health, practitioners and researchers across the world have been developing and researching arts inventions. The focus of this research has been on activities that not only facilitate the exploration of creativity but are also enjoyable, accessible and have significant impact upon key health indicators (MacDonald, Kreutz, & Mitchell, 2012a). In many ways, research within the musical domain has been at the cutting edge of this new generation of research investigating the beneficial effects of arts participation. One of the key drivers in this vigorous research milieu is the growth of qualitative research within health care contexts and researchers interested in exploring the potential benefits of musical participation have fully embraced the advances that have taken place in health-related qualitative research. Much of the qualitative research discussed within this article utilized phenomenology as a key theoretical approach (Moran, 2000). In particular, interpretative phenomenological analysis (IPA) (Smith, Flowers, & Larkin, 2009) is utilized as an analytical framework. IPA is particularly useful in the context of music and health as it has a focus on personal lived experiences and how participants make sense of their experience. A number of different types of qualitative research projects are presented and these focus on exploring the process and outcomes of music interventions. This article also presents a new conceptual model for music, health, and well-being. This new model develops on a previous version of MacDonald, Kreutz, & Mitchell (2012b) by incorporating new elements and contextualization and providing detailed empirical examples to support the various different components. The model is also developed with the aim of increasing multidisciplinary dialogue across the multitude of professions that are involved in researching the relationship between musical participation and wider health parameters. With music therapists at the vanguard, this group of professions includes psychologists, neurologists, teachers, occupational therapists, medical doctors, and architects. This article also addresses an urgent need for cross-pollination of ideas and collaborative research projects incorporating multidisciplinary dialogue across all disciplines involved in researching the relationship between music, health, and well-being. After an overview of the model, this article presents a number of empirical examples to further highlight both the distinctive features and the overlapping elements of the various disciplines involved in practising within the overarching topic of music, health, and well-being.

## Music therapy

**Figure 1:**
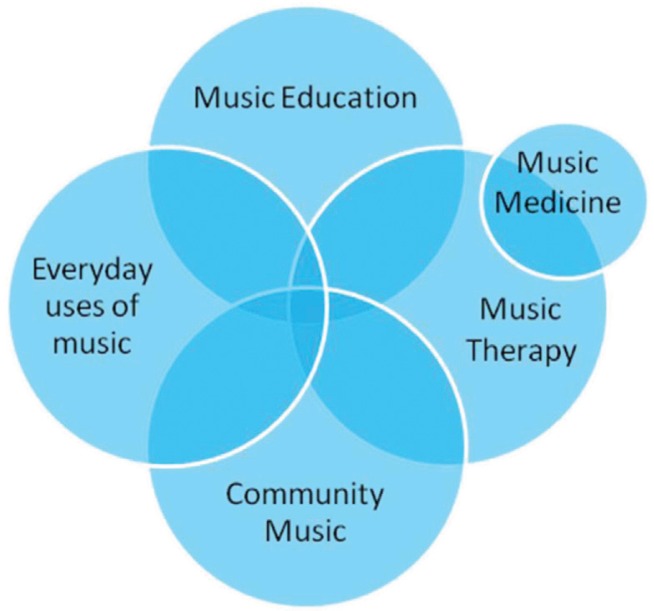
Conceptual framework for music, health and well-being.

When conceptualizing the entirety of interventions that are defined within the music, health, and well-being framework, there are a number of discrete but related areas that can be considered, and these are outlined in Figure 1. The first is music therapy, and it is important to acknowledge that while the field of music, health, and well-being is currently experiencing significant interest, the discipline of music therapy has a long history of research dating back to the early part of the 20th century (Bunt, 1994). Indeed, the profession of music therapy within a modern context has been developing practice and producing research for nearly 100 years (Wheeler, 2009). This work has had a significant impact and there are a number of well-established journals dedicated to research within the area of music therapy (Bonde & Trondalen, 2012). Music therapy has many different definitions but for the purposes of this article, a key element of the music therapy process is an emphasis upon the therapeutic relationship between clinicians and clients or participants. Thus, music interventions that fall under the music therapy category will focus on positive psychological and/or physiological benefits for the participants and the interventions will be delivered by qualified music therapists. Also, music therapy interventions neither will have musical developments in terms of increasing technical skills as a primary objective nor will they be primarily concerned with a general increase in artistic activities within the musical domain. For a comprehensive review of music therapy approaches, see Bonde & Trondalen (2012). Recent advances in music therapy included new models of practices that incorporate community-based activities (Ansdell, 2004; Stige & Aarø, 2011). In particular, community music therapy is one example (Pavlicevic & Ansdell, 2004; Stige & Aarø, 2011). The concept of health musicing also broadens the music therapy approach to include a multitude of activities outwith the conventional clinical context (Ruud, 2012; Stige & Aarø, 2011).

## Community music

Community music in contrast will not have therapeutic effects as a primary concern but may have increased access to artistic activities outside conventional institutional setting as an objective (Hallam & MacDonald, 2008; Higgins, 2012). Community choirs and percussion classes are good examples of community music interventions. Also, there may not be an emphasis on the development of discrete technical skills but the primary objective may be providing an opportunity for creative expression in informal settings (Veblen, Messenger, Silverman, & Elliott, 2012). However, and this is an important point, many community music interventions view positive psychological benefits as an important secondary benefit (Hallam & MacDonald, 2008). For example, a community choir may be seeking to give older adults the chance to enjoy singing together but the enjoyment, freedom of expression, and social support afforded by a choir may bring about developments in self-confidence and self-esteem. Thus, there is an overlap between community music interventions and music therapy interventions. Also, the recent developments in music therapy, discussed above, have reached into community contexts (Pavlicevic & Ansdell, 2004). In these situations, music therapists may facilitate music groups in informal settings and while the aims may still be explicitly therapeutic, the social and musical context may have more in common with community music interventions that what might be considered as a traditional music therapy context (hospital, health centre, private practice, etc.).

The field of community music is an exponentially growing area of interest for music researchers and over the past 15–20 years, the increasing output of journal articles has supported its development into a distinct and unique field of practice (Higgins, 2012). It is now an established area of research with a dedicated journal, *The International Journal of Community Music* and a commission of *The International Society for Music Education*. Although it appears to have significant and distinct characteristics, it is an area that is difficult to define as there are many overlaps into “general” areas of musical activity. There are many descriptions of the term “Community Music.” On the one hand, it is practiced throughout the world in formal and informal settings and in some respects all music making can be defined as “community-based” since all music has a social context. However, community music has emerged as a distinct field of practice and as an influential approach to both music education and as a means of increasing access to all types of musical activities. Indeed, a key concern of community music practitioners is to increase access to music making for all members of the public (Veblen, 2008).

Definitions of community music focus upon the practical, activity-based features of community music. They also discuss fluid hierarchies that may exist within community music activities and these definitions also emphasize the process-based nature of community music. The published literature discusses educational issues relating to the training of community music practitioners and also the educational emphasis of community music programmes (Higgins, 2012). Finally, many of the published papers within community music discuss the wider benefits of community music activities. These benefits may be for the individual but they also extend to the group and in some cases reach out further to resolve conflicts and develop empathy between different groups. For example, Bowman (2009) discusses how musical engagements in community music settings develop “character, habits, dispositions.” Large-scale community music groups like *El Sistema* have come to international attention over the past 10 years. Founded in 1975 by economist and musician José Antonio Abreu, El Sistema is a publicly financed voluntary sector community music education programme in Venezuela which provides access to music education for thousands of children from disadvantaged backgrounds. There are two significant El Sistema projects running in Scotland, UK. In a group setting, Langston and Barrett's (2008) case study of a community choir illustrates the value of the “social capital” that is derived from being a member of the group and for conflict resolution beyond the group, Hayes’ (2007) report notes how a community music group (in this case a choir formed from gay/lesbian/bisexual/transgender communities) can help bind communities by “building bridges through song.” While all these interventions are undoubtedly community music in their design and delivery, the therapeutic aims and speculated outcomes create some overlaps with music therapy and this is represented in the overlapping circles in Figure 1.

## Music education

Music education is another key element of Figure 1 and recent advances now mean that music education has much to contribute to the music, health, and well-being agenda. In most contexts, music education is defined by an explicit focus upon the development of conventional music skills. For example, many music classes in schools and universities focus on developing an instrumental technique or specific technical knowledge. Private lessons may involve pupils being taught by a teacher with the goal of passing grade exams. Once again the primary function is not therapeutic or social, however, many music educationalists are interested in the wider benefits of music teaching. Indeed, recent research has begun to investigate the effects of conventional music lessons upon other non-musical aspects of psychological functioning and here there is an overlap with music therapy and community music (Butzlaff, 2000). For example Costa-Giomi (2012) examines the evidence to support the assertion that attending music lessons can produce significant increases in other cognitive areas. Raucher (2008) also discusses the possible beneficial effects of music education upon a range of psychological and social variables. There has also been considerable debate about the effects of music upon a range of cognitive skills that may be enhanced via listening (Johnson & Memmott, 2006). However, there is significant discussion within the academic community about the extent to which these effects are reliable and to what extent they sustain over time and there is currently no way to predict the precise effects of music listening on cognitive functioning (Schellenberg, 2012).

Also, the revolution that has taken place within music education over the past 20 years means that school and university music education is no longer dominated by western classical music. It is now possible to study popular music and engage in more informal types of music activities within an institutional music education framework. Thus, music education now has overlaps with community music as well as with music therapy. Indeed, the growth of community music over the past 20 years in many ways echoes the revolution that has taken place in music education. While music is one of the most important recreational activities that young people engage with (Zillmann and Gan, 1997), there is evidence to suggest that adolescents lose interest in formal music education at just around the same time that music is becoming a crucial part of their identity (MacDonald, Miell, & Hargreaves, 2002). The movement within music education to broaden its scope and remit to include a wider range of music experiences, incorporating popular music, has resulted in a regeneration of formal music activities within educational institutions. This move is partly responsible for the increased interest in music, health, and well-being within music education.

## Everyday uses of music

The 4th segment of Figure 1, everyday uses of music, is not a distinct field of practice in the way the other sections are; however, it has significant relevance to the debate around the effects of music on health and well-being (DeNora, 2000). Continuing research within the psychology of music has highlighted the profound effects of music listening and there is no doubt that music is a separate channel of communication affecting emotions in significant ways (Hargreaves, Miell, & MacDonald, 2012). Music may be uniquely suited to managing or regulating emotions and stress in everyday life since it has the capacity to both distract and engage listeners in a variety of cognitive and emotional ways (DeNora, 2010; Mitchell & MacDonald, 2012; Saarikallio, 2011; Sloboda & O'Neill, 2001). Every time we select a piece of music to listen to, we make a number of very sophisticated and highly nuanced psychological assessments about our current state of mind and the environment in which we are listening to music. For example, *how do I feel right now, how do I want to feel in five minutes, what music will help me achieve these goals. Who else is listening*? *What they will think of my musical choices?* Importantly, these complex psychological assessments are made quickly and in many ways without explicit conscious effort. In this way, we recognize that our musical listening has profound effects on how we feel and also affects the other people who may be listening to our musical selections. Music listening is therefore crucially implicated in mood maintenance and we can think of our music selection as a form of psychological self-help. Beneficial effects of music listening on subjective well-being and physical health outside clinical contexts have been reported by a number of researchers (DeNora, 2007; Pelletier, 2004; Standley, 1995). This issue has become even more important as modern technological advances mean that we can now listen to our own personal music collection 24 hours a day. A key point is that informal music listening may have significant positive effects upon our health and well-being and there is a growing recognition that this is now an important field of study. Thus, work within the everyday uses of music category has overlaps with music therapy. Also, everyday music listening has connections with music education in the sense that our music preferences influence how we may want to learn to play a musical instrument and music listening forms an important part of many formal music education programmes. Everyday music listening also overlaps with community music in many ways in that our musical taste and listening habits inform decisions about how we may wish to engage in music making. These types of disciplinary overlaps are explicitly incorporated into the model highlighted by the overlapping circles.

## Music medicine

The final section of the graph, music medicine, refers to a specialized area of work within music, health, and well-being taking place within medical contexts. It is perhaps a more focused and specialized discipline with fewer people working in this area than in the other broader categories of the model. However, it can be considered a distinct field of practice with textbooks, journals, and definitions of the type of work that takes places with this area (Spintge, 2012). The work of Ralph Spintge has been particularly influential in developing the practice of music medicine. A typical type of music medicine intervention may involve patients undergoing operations listening to music to help reduce pain and anxiety perceptions. This is based on the observation that patients undergoing medical treatment in hospital operating theatres suffer from complex sets of conditions including pain, anxiety, and distress and that music listening may offer an opportunity to ameliorate these symptoms. There is a growing body of evidence highlighting the positive effect of these types of music medicine interventions upon both psychological and physiological parameters. On-going research in this area now spans 3 decades and 160,000 participants (Spintge, 2012). Various different types of methodologies have been used to develop this area of work including psychological and physiological measurements such as self reports (open questionnaires, Thematic Apperception Test, e.g. Westen, 1991), observable behaviour and facial action coding systems (Ekman, 2003), plasma levels of stress-hormones, EEG, PET, neurovegetative and cardiovascular responses, drug consumption, length of hospital stay and other economic outcome variables (Hatano, Oyama, Tsukamoto, Sakaki, & Spintge, 1983; Spintge, 1992). This approach is applied in surgery and anaesthesia, dental care, pain medicine, palliative care, intensive care, obstetrics, paediatrics, geriatrics, ophthalmology, and neurology (Arnon et al., 2006; Brice & Barclay, 2007; Leard, 2007; Leins, 2006).

In some ways, music medicine interventions are very closely related to music therapy as they explicitly have therapeutic outcomes as a primary objective. They have no overlap with music education or community music hence their position within the model. Another important point is that most music medicine interventions use “prescribed music” and so clinicians make informed assumptions about the effects of particular pieces of music upon the patients’ psychological and physiological functioning. The relationship between structure and preference is a key point discussed in more detail below.

A central aim of this article is to highlight the parallels and contrasts between the different sections of the model and to further develop multi-disciplinary dialogue between professional practises that are quite distinct, but also have significant points of overlap. For example, community music therapy can take place outside institutions such as hospitals and schools and therefore have elements in common with community music (Stige & Aarø, 2011). Also, some of the aims of community music therapy may overlap with community music so both interventions may share the goal of increasing access to music activities for disadvantaged groups. Thus, the intersection between community music and community music therapy within the model becomes an important area of overlap. Similarly, community music and music education may also share some goals and employ similar practises (e.g. the development of instrumental skills through teaching). Thus, the two circles representing music education and community music can also overlap in important ways. Music education can include wider psychological developments as a secondary goal and this can overlap with therapeutic approaches. Everyday music listening also has important intersections with the other components of the model. Individual music preferences can be an important part of music therapy interventions and formal music education takes into consideration students’ tastes and preferences when developing curriculum guidelines. Similarly, community music approaches have everyday music listening, tastes and preferences at the heart of interventions that seek to increase access to music activities by developing enjoyable and rewarding music projects for participants. There are many ways in which all the major components of the model can overlap so the section at the centre is also important. For example, community music interventions that have musical developments and psychological developments as an aim that also focuses on compositional activities, will include elements of all the large four circles within the model and so will occupy the intersections of the circles at the centre of the model.

## Empirical examples

The following section of this article gives some empirical examples highlighting how related research covers the various categories within the model. This work further shows how the different categories have discrete characteristics but also overlaps with the other areas. These examples also signal the importance of qualitative methods in developing knowledge regarding the process and outcomes of music, health, and wellbeing-related interventions.

## Music therapy

Pothoulaki, MacDonald, and Flowers (2012) report the results of a music therapy intervention investigating the relationship between the categories of community music and music therapy. In this study, nine patients at a cancer hospice received group music therapy focused on improvization, once a week for 12 weeks. Before and after the intervention, all participants were interviewed. These interviews were transcribed and analyzed using IPA music as the analytical framework work (Smith, Flowers, & Osborn, 1997). The analysis revealed a number of key themes namely; *playing the instruments, group interaction/dynamics, self-confidence, relaxation: haven, escape and being carried away, stress relief, the importance of the group, positive feelings and the musical experience, illness-forming a strong bond, free expression-communicating through music*.

The extract below is an example from the free expression and communication through music category:

Yes. Well, we can all communicate (.) at the same time (.) by playing an instrument whereas if you are verbally communicating you cannot all talk at the same time, whereas we can all play a tune and all be heard at the same time. And then if you hear someone, you can pick up their rhythm and you can join in as well or maybe pick up someone else and join in with them. So, everybody is playing a tune and everybody is communicating and you can pick, (.) you know, certain tunes or sounds (.) or rhythms if you like and join in with the other person.

This participant is emphasizing how music functions as a separate channel of communication. Importantly, for this participant, as was the case for many of the participants, a belief that music was free from the constraints of the rules of spoken language-facilitated freedom of expression that was viewed as crucial to the process and an important part of why the sessions had therapeutic value. A key point here is that while this intervention is explicitly a music therapy approach, the group improvization sessions were set up and delivered using ideas that also resonate with a community music approach. Participants all defined themselves as non-musical and the sessions were designed as an opportunity for participants to explore their creativity and have fun in a friendly environment while giving access to creative music activities.

Another type of music therapy example of relevance is Therapeutic Songwriting. Therapeutic Songwriting is a music therapy intervention where participants compose new music as a means of tackling health-related problems (Baker & Ballantyne, 2012). Central to this approach is a creative collaboration between the client and therapist within the context supportive therapeutic relationship (Wigram & Baker, 2005). There is growing evidence to suggest that this type of work leads to positive therapeutic outcomes across a range of diagnoses (Baker et al., 2008). Therapeutic Songwriting creates opportunities for people to develop, negotiate, and maintain many different types of identities. Moreover, participants are able to construct life narratives through song and this can facilitate personal reflections on key aspects on life; relationships, beliefs, qualities and attributes, and so on (Baker & Ballentyne, 2012; McFerran, Baker, Patton, & Sawyer, 2006). Group identities can also be explored through these types of compositional activities (McFerran et al., 2006; McFerran & Teggelove, 2011).

Baker and MacDonald (in press) report the songwriting experiences of 26 participants involved in a therapeutic songwriting project with a qualified music therapist. Participants were interviewed about their experiences after the creation of each song and again at 6-week follow-up. The analysis highlighted five main themes: *artistic concerns; initial expectations; responses to listening to one's own song creations; exploring the self; and relationship with the therapist*. Flow experiences of the type described by Csikszentmihalyi (1998) were evident in the transcripts. Importantly, these experiences have been highlighted by many researchers as being key indicators of positive and rewarding experiences during artistic endeavours (MacDonald, Byrne, & Carlton, 2006). Flow experiences of being fully immersed in the songwriting process, altered perception of time, and experiencing a balance between ability and effort were especially evident in people's description of their creative processes. Song writing was viewed as an enjoyable means to explore the self, enhancing mood, and creating a satisfying artistic product. Flow experiences were evident in participants reporting of changes in perception of time. Time was perceived to have moved fast; participants sensed they “lost track of time” and were disappointed when their sessions came to an end.

I just find time passes so fast. I looked at my clock as you went out, and it had been an hour. I was like, ‘are you serious?’ It felt like it was only like fifteen minutes.

As participants created songs, they experienced becoming fully focused. They described being “fully absorbed and in tune”, unaware of other things going on around them in the environment, “everything else seemed to disappear”.

I was just speechless. It was breathtaking. It was a really intense euphoria I guess I was just so lost in the music.

I just felt totally into it. It really absorbed my full conscious, totally just focused you know on writing a good lyric or verse …

The above research projects are good examples of music therapy interventions that overlap into community music. For example, Therapeutic Song writing challenges the existing stereotypes regarding the composition process. Traditional views of composition construct this activity as an elite musical process and one that can only be seriously undertaken after intense musical study. However, the results from this work highlight that individuals with little music experience can engage in compositional activities that can be meaningful, rewarding, and enjoyable. Similarly, the music therapy with cancer patient example also shows how individuals who self-define as non-musicians can engage in group improvization sessions that are musically and artistically rewarding. Both of these examples emphasize the universal potential of music communication within everybody and highlight the overlap between community music and music therapy interventions. Importantly, one distinctive feature that separates these music therapy approaches from the others presented in this article is that the music therapist forms a clinical relationship with the participants and this clinical relationship is of paramount importance within the musical environments. These projects also have links with music education approaches since Therapeutic Songwriting may also facilitate the development of compositional skills and confidence in music making. Similar developments in confidence may also be evident for the participants in the improvization study who may also develop instrumental skills. Finally, this work also has connections with the music in everyday life section of the model since musical preferences and listening histories are crucial influences when composing new songs.

## Everyday music listening

While everyday music listening is not a distinct field of practice in the same manner that the other sections of the diagram operate, it is still a crucial element of the processes related to music, health, and well-being interventions (Skånland, 2011; Skånland, 2012). One of the principle reasons we listen to music is for mood regulation and in this way music can be considered an informal type of self-medicated therapy. Two important and related issues must be taken into consideration when investigating contemporary music listening practices. The technological revolution in relation to digital listening devices has facilitated access to entire personal music collections via small digital devices 24 hours a day (MacDonald et al. 2012b). Also, increased understanding of how personal and emotional factors beyond the music itself influences music perception and highlights the importance of the associative context of listening in terms of predicting the effects of music (Juslin & Sloboda, 2010; MacDonald et al. 2002; Miell, MacDonald, & Hargreaves, 2005). The importance of familiarity and past associations makes understanding the role of personal preference when investigating the effects of music crucial. Two studies demonstrated that preferred music could be an effective aid to reducing anxiety for patients in hospital contexts (MacDonald et al., 2003). A further study in a Greek hospital with patients undergoing going kidney dialyses reported a significant reduction in pain perceptions for participants listening to self-selected music during the procedure (Pothoulaki et al., 2008).

Further examples of work in this area include a series of studies investigating the effects of preferred music upon a variety of psychological variables. In these studies, participants listened to self-selected music during a perceptual experiment. Results showed positive effects upon pain and tolerance levels in laboratory settings where participants listened to different types of experimental stimuli while undergoing the cold presser technique (Mitchell, MacDonald, & Knussen, 2008). This technique invites participants to place their hands in cold water until the water becomes too cold.

In the first of these studies (Mitchell, MacDonald, & Brodie, 2006a), 54 participants selected recorded music from personal collections. During a listening experiment, participants tolerated a painful stimulus significantly longer and reported feeling significantly more control over this pain when listening to their preferred choice in comparison with white noise or conventional “relaxing music.” A further study compared visual and auditory stimuli using a similar design (Mitchell, MacDonald, & Brodie, 2006a). This study compared the effects of self-chosen music to a selection of 15 well-known paintings, and to a silence control in 80 participants. Additionally, a measure of state anxiety was taken following each condition and a music listening behaviour questionnaire investigated everyday listening habits. Findings of this study largely replicated the earlier results; preferred music listening leading to significantly longer tolerance and greater perceived control than both silence control and chosen art. Anxiety, measured by a short-form state anxiety questionnaire (Spielberger, 1983) was significantly lower during music listening compared to the other two conditions. The aim of the musical behaviour questionnaire was to explore individual differences in music listening and to help identify who may benefit most from self-selected music listening. Those people who listened to favourite music most frequently experienced significantly lower levels of anxiety. This suggests that familiarity may indeed be important in this therapeutic context, potentially combining anticipation and tension release from the musical flow and structure itself with the emotionally engaging associations held with it. A further significant correlation was found between knowledge of lyrics of the chosen song and pain tolerance during the music condition, again supporting the importance of familiarity in engagement.

In terms of the underlying psychological mechanisms, listening to self-selected music may induce heightened emotional response and distract attention more effectively. Concurrently, selecting and listening to one's chosen music may facilitate a sense of increased control in unfamiliar or threatening situations. Music listening in these contexts acts as a stimulus that is distracting. When we are listening to our favourite music, we are not attending to the noxious stimulus or at least attending to it with less intensity. Listening to our favourite music is also emotionally engaging so this emotional engagement draws us away from the noxious stimulus. Also, listening to self-selected music in a laboratory context represents bringing a familiar stimulus into an unfamiliar environment and may also enhance feelings of control in unfamiliar situations.

A key question raised by these studies is to what extent the structural features of the music (e.g. tempo, mode, rhythm etc) were important in facilitating the therapeutic effects. The music selected by participants across the these studies cover a wide range of genres, including chart pop, rock, punk, hip-hop, and dance. Examples of opera (Pavarotti), metal (Metallica), pop music (The Beautiful South) were evident. These varying styles of music, with many different structural features, yielded similar effects of increased pain tolerance and perceived control. Therefore, music with very different structural features can be rendered functionally equivalent by the role of preference. That is, the participants chose music with contrasting structural features to produce the same effect, namely, reducing pain and anxiety perceptions. Specifically, key aspects of the therapeutic potential of music listening relate to an associative relationship that a listener has with a piece of music, for example the music may remind the listener of a happy occasion.

To further investigate this issue, Knox et al. (2012) performed detailed structural and content analysis of a selection of tracks found to be effective in these experiments. From three of the previous studies (Mitchell et al., 2006a; Mitchell, MacDonald, & Brodie, 2006b; Mitchell & MacDonald, 2009), 76 tracks were selected as having the greatest overall effect on pain perception. Following structural analysis of the music, mood classification results showed that preferred music chosen by participants fell predominantly in the “content” mood cluster as defined in the *Circumplex Model of Affect* by Russell (1980). This indicates a tendency towards low arousal and positive valence. The results suggest that in addition to personal preference, emotion expressed by music, as defined by its acoustical content, is important in enhancing emotional engagement with music and therefore some structural features are important in predicting therapeutic effects. This result indicates that pain tolerance was greater for music that has less tonal (pitch) variation. This result suggests that pain intensity levels are lower for music in which there may be less prominent chord changes, bass lines, or strong melodies. See Knox et al. (2011) for a full discussion of key structural features.

This discussion also has relevance to music medicine interventions because, as stated above, music medicine interventions place particular emphasis upon the structural features of music and relationship between these structural features and therapeutic outcomes.

In summary, preference is a key variable to take into consideration when investigating the therapeutic effects of music listening. Preference interacts with structure in such a way that when participants are free to select their favourite music, they choose music with certain structural features that facilitate this therapeutic effect. This approach combines some of the key features of music listening in everyday contexts with a music therapy approach and can therefore be considered as operating in the area between everyday music listening and music therapy.

## Community music

There is a growing body of research investigating the processes and outcomes of community music and one particular set of studies highlights the overlap between music education, music therapy, and community music (MacDonald, Davies, & O'Donnell, 1999). This project investigated a music intervention focused on playing a Javanese Gamelan. The intervention was delivered by a Glasgow-based music production company called Limelight (previously Sounds of Progress) who specialize in working with disadvantaged groups and individuals with special needs. A key feature of these studies is that the aim of the workshops was not primarily to teach people music or to deliver positive therapeutic effects for the participants. The primary aims of the Gamelan workshops were to increase access to enjoyable creative music activities for individuals from disadvantaged groups; in this case a group of individuals with mild or moderate learning difficulties. However, a secondary goal of the intervention was the development of specific music skills for the participants. Furthermore, it was also hypothesized that the participants would develop along a number of psychological dimensions. Thus, this community music intervention also had educational objectives (the development of music skills) and therapeutic objectives (psychological developments). In comparison to a number of control groups, the 20 individuals who attended 1 hour Gamelan workshops once a week for 3 months showed significant improvement in musical ability and communication skills. Moreover, the communication improvements correlated with the musical improvement scores. A subsequent qualitative study investigated the subjective experiences of individuals who took part in similar activities organized by the music company. The qualitative interviews highlighted the importance of these types of community music activities for developing positive music identities, once again highlighting the interplay between community music, music education and therapeutic outcomes. The two extracts below emphasize the importance of professional standard music activities for the participants

T: it makes it easier, sometimes, to talk to people, yeah (.) you know, having a, having a gift (.) because (.) at one time (.) people would come up and talk to my Dad instead of me, and (.) but now when they hear you singing […] you know they'll come and talk to you. […] It was great when *SOP* did the school tours, remember the school tours?

I: aye [interviewer was involved with this project]

T: it was great the kids never treated (.) the kids came up and asked for your autograph you know, just the same as an ordinary (.) an ordinary, just the same as they would (.) you know

T: I remember I used to go up in the ambulance up to the hospital years ago (.) and there was this old woman she was always complaining about her illness (.) we used to call her 57 varieties! (both laugh) She used to always say about me, ‘you know, he's in a wee world of his own there’ (.) and you're sitting listening! (both laugh) and you're sitting listening ‘oh aye, I'm in a wee world of my own here!’ (laughs) (.) but there again, (.) that same old woman, I started a sing-song in the ambulance one time and she started to talk (.) she started to talk to me normally! (laughs) you know what I mean? (both laugh) so there you go […] she forgot about the ‘wee world of my own’ when I started the sing-song! […] The attitude changed.

In both extracts, the participant points to an important change in the difficulties he experienced in interacting with others was brought about by his music. When his identity as “musician” became salient to them, rather than seeing him only as “disabled,” people began to relate to him directly rather than ignoring and bypassing him. In the first extract, this was in a professional context in which the audience asked him for autographs as they would of any performer, and although the other was not a professional context, music again served the purpose of facilitating interaction. The first extract highlights a very common and well-reported issue for individuals with disabilities; that they are often ignored in public situations.

The use of the terms “ordinary” and “normally” in the examples above is important since they underline the point made by many disability researchers that to be seen and treated as “ordinary” and “normal” is so often to be seen as something different from having a disability. An important struggle for many people with impairments in dealing with other people's expectations is to extend the use of the terms “normal” and “ordinary” beyond the able-bodied community, which is so often what those terms imply. For the participants, their perceptions of the shift in people's assumptions and expectations following a music performance were striking. The extracts identify some important and pervasive themes for understanding the powerful role which music can play in this process.

The qualitative study explores the social roots of personal identity; examining the details of complex interactions between individuals involved in musical activities and the reflections on these interactions by some of the individuals involved, in particular the impact of such activities on their changing personal identities. Musical activities can be particularly effective as catalysts for identity development because of the high degree of mutual engagement necessary between performers, and because of the impact of being involved in valued activities on their feelings of self-confidence and empowerment. Once again, signalling the interaction between therapeutic and educational outcomes within a community music context.

The results from the quantitative studies highlighted the impact that music interventions can have on discrete personal and social factors. The qualitative examples suggest that involvement in musical activities also has more general effects on the way in which people think about both themselves and their position within society. These two developments are related in that music can be thought of as not only facilitating specific changes in musical and psychological factors, but also as contributing to the identity projects in which the individuals are engaged. Whilst the above examples focuses our debate upon the activities of one particular music company, this has been presented as an example of how any musical participation, suitably structured, can be an excellent vehicle for leading to musical and personal gains for participants. These effects will not only be found with participants in *Limelight* activities, but rather suggest that when music is employed for therapeutic/educational objectives in a structured and goal-directed way by individuals with musical expertise and training, then outcomes of the type reported here can be expected.

As one participant explains in the extract below, he has made profound and fundamental developments in personal identity through his involvement in musical activities, and it is important to explore the sites of such changes as well as individuals’ reflections on these processes. For this participant and others, working with others in the professional theatre and music world was in some senses awe-inspiring, and yet also made possible the identification of common efforts and ambitions amongst all in the show, transcending their other differences, and giving them a common identity as professional musicians and actors.

T: when I worked with (.) when I did that show with Wildcat [well known Scottish professional theatre company] you know, you really felt ‘oh god, you're working with all these you know (.) (laughs) people’ you know

I: uh huh

T: you know, it makes you feel (.) makes you feel (.) you must, you must have been, (.) you must have been worth something, you know (unclear) it's better not to get (.) you know too big headed about it, Gordon

I: aye

T: but you just feel, you feel different, you're working with these people you know – (laughs) you're probably part of these people (laughs) when you're in a show with them!, you know! But they're just the same, (.) they're no different from anybody, they're just the same as you and me

## Discussion

The preceding paragraphs have presented a number of overarching themes. First, a model outlining how music, health, and well-being can be conceptualized as a distinct field of practice was discussed. The unique features of the different elements: music therapy; music education; community music; music in everyday life; and music medicine were emphasized but important points of overlap between the areas were highlighted as crucial points of multidisciplinary exchange and collaboration. These intersections of practice are particularly important areas of interest in terms of developing future research agendas. This article then presented a number of experimental examples highlighting the parallels, contrasts, and elements of overlap across these areas.

Two projects utilizing an explicit music therapy approach were discussed. The first, a group improvization session with cancer patients, highlighted key features of the musical and social environment that can facilitate positive experiences for the participants. A key point here is that the improvisatory context of the sessions was of paramount importance. Improvization in general and improvized music specifically is currently the focus of considerable academic attention (MacDonald & Wilson, in press). Musical improvization provides opportunities for negotiating difference through creative collaboration and understanding the unique musical, mental, individual and social processes through which improvization takes place in music is, a key area of interest for music psychology (Wilson & MacDonald, 2012). The unique psychological and musical features of improvization make it an important artistic, educational, and therapeutic process and as such has considerable relevance for the model presented within this article. Improvization is also accessible in that it is a process that everybody can engage in; we are all musical improvizers at some level (MacDonald & Wilson, in press).

The second study within a music therapy context utilized Therapeutic Song writing as an approach. This project highlighted ways in which the song writing process can be creative, collaborative, and expressive and an important technique for facilitating positive developments for participants. These elements link both the music therapy research projects and also relate to other projects presented in this article. For example, research presented within the community music section of the article focused upon the work of *Limelight* and this example highlighted developments in discrete psychological and musical variables and also developments in identity processes for participants. Key elements of the musical social environment within this project included a professional approach to music making and the opportunity for performance and recording activities. Thus, the music therapy examples and the community music examples share key musical and social elements with each retaining distinctive features. All of these examples focus on increasing access for creative music activities for groups of individuals who can be considered “disadvantaged.” All of the interventions take an inclusive egalitarian approach to music making that allows participants considerable freedom to develop musically according to their needs and goals. These processes are scaffolded within a safe and encouraging environment by expert musician(s) or therapist(s) who help facilitate the process using their expertise and knowledge. The music therapy inventions will retain a distinctive focus upon the therapeutic relationship between client and participants and the community music project will retain a focus upon the development of basic music skills.

Another important focus of this article is to highlight the utility of qualitative methods for developing knowledge of the process and outcomes of music interventions focused upon health and wellbeing. Qualitative methodology has a number of key features that make it a particularly useful approach to research within this area. Music is completely woven into the fabric of our lives. It provides the focus of, or the soundtrack to, countless social situations, and can also provide refuge or solace in private moments. Also, everyone has a sense of the ways in which they participate in music, and of their own level of ability and interest, and so it forms an important part of identity development and maintenance for many people, especially given that music plays a central role in contemporary society (MacDonald et al., 2002). Thus, qualitative methods, which are particularly good for giving a voice to the subjective and phenomenological aspects of experience, can help reveal the unique ways in which music is important within the lives of individuals. Also, qualitative methods provide ways in which commonalities across individuals’ different musical experiences can be developed and synthesized. For example, in Pothoulaki et al. music therapy study previously discussed, all of the participants self-defined as non-musicians yet each had highly nuanced and individualized musical identities that were important during the improvization group sessions. IPA, as an analytical framework, enabled these individualized music identities to become manifest in the analysis and for key themes to emerge. One conclusion from the analysis was that the musical communication was enjoyable and meaningful for all of the participants. Also, this musical communication had important and tangible connections to particular aspects of their lives.

This discussion raises a number of issues about professional practises and identity for the practitioners involved. All of the professionals whose work has relevance to music, health, and well-being have highly developed and specific skill sets that enables effective working within each of the areas. Music therapy in particular has a very diverse and highly specialized range of different types of music clinicians working under the general heading “Music Therapist” (Ansdell & Pavlicevic, 2008; Barrington, 2008). For example, a psychodynamic dynamic music therapist will practise in very different ways from a community-based music therapist. There are now different models of health musicing and these different models all have different types of interventions associated with them (Bonde, 2011; Ruud, 2012).

## Conclusions

This article has explored music, health, and wellbeing across a range of contexts. Using the categories of music therapy, music education, music medicine and music and everyday life, it highlights how the current research into the beneficial effects of music has universal relevance across all areas of health and social care and incorporates topics from across the academic spectrum. Moreover, the multidisciplinary relevance of this topic facilitates a pluralistic approach to research and practice that incorporates theoretical ideas from each of the disciplines presented. It is important to note that these disciplines contain highly distinct features whilst simultaneously having areas of overlap and points of intersection as demonstrated by the conceptual model. While there is still much to learn about the process and outcomes of well-being-related music interventions, it is a field of practice and research that has major contributions to make in positively influencing key aspects of health. Qualitative research methods have much to contribute to this process since these approaches facilitate the exploration of the subjective and phenomenological aspects of musical experience and it is these very individual aspects of musical life that lie at the heart of why music has powerful beneficial effects upon health.
